# Impfung zur Vorbeugung der COVID-19-Erkrankung sowie Impfpriorisierung bei Epilepsie

**DOI:** 10.1007/s10309-021-00404-5

**Published:** 2021-02-01

**Authors:** Adam Strzelczyk, Susanne Knake, Martin Holtkamp, Andreas Schulze-Bonhage, Johannes Lemke, Sarah von Spiczak, Ralf Berkenfeld, Felix Rosenow, Christian Brandt, Friedhelm C. Schmitt

**Affiliations:** 1 Deutsche Gesellschaft für Epileptologie e. V., Reinhardtstr. 27c, 10117 Berlin, Deutschland; 2 „DGN Kommission Epilepsie und Synkopen“, Deutsche Gesellschaft für Neurologie, Berlin, Deutschland; 3 Epilepsiezentrum Frankfurt Rhein-Main und Zentrum für Neurologie und Neurochirurgie, Goethe-Universität Frankfurt, Schleusenweg 2–16, Haus 95, 60528 Frankfurt am Main, Deutschland; 4 Epilepsiezentrum Hessen und Klinik für Neurologie, Philipps-Universität Marburg, Marburg, Deutschland; 5 Epilepsie-Zentrum Berlin-Brandenburg, Klinik für Neurologie, Charité-Universitätsmedizin Berlin, Berlin, Deutschland; 6 Epilepsiezentrum, Neurozentrum, Universitätsklinikum Freiburg, Freiburg, Deutschland; 7 Institut für Humangenetik, Universitätsklinikum Leipzig, Leipzig, Deutschland; 8 DRK-Norddeutsches Epilepsiezentrum für Kinder und Jugendliche, Kiel-Raisdorf, Deutschland; 9 Neurologische Praxisgemeinschaft, Neukirchen-Vluyn, Deutschland; 10 Epilepsie-Zentrum Bethel, Universitätsklinik für Epileptologie, Krankenhaus Mara gGmbH, Bielefeld, Deutschland; 11 Universitätsklinik für Neurologie, Otto-von-Guericke-Universität, Magdeburg, Deutschland

**Keywords:** Status epilepticus, Epileptischer Anfall, SARS-CoV‑2, Corona, Pandemie, Status epilepticus, Seizure, SARS-CoV‑2, Corona, Pandemic

## Abstract

Der Vorstand der Deutschen Gesellschaft für Epileptologie und die Kommission „Epilepsie und Synkopen“ der Deutschen Gesellschaft für Neurologie haben die aktuelle Datenlage zur Impfung zur Vorbeugung der Corona-Virus-Krankheit 2019 (COVID-19) sowie zur Impfpriorisierung bei Menschen mit Epilepsie gesichtet, diese zusammengefasst und geben die unten genannten Empfehlungen ab.

## Abstract (en)

The Board of Directors of the German Society of Epileptology and the committee on epilepsy and syncope of the German Society of Neurology have reviewed the current data on vaccination to prevent coronavirus disease 2019 (COVID-19) and vaccination prioritization in people with epilepsy and provide a summary and recommendations.

Seit Dezember 2019 wurden erstmals in Wuhan (China) Erkrankungen mit dem neuartigen Corona-Virus SARS-CoV‑2 beschrieben, die sich mittlerweile zu einer weltweit umspannenden Pandemie entwickelten. Die Erkrankung durch das „SARS-CoV-2“-Virus manifestiert sich im Regelfall als Infektion der Atemwege und wird als „COVID-19“ bezeichnet. Zu den häufigen Symptomen gehören Fieber, Husten und weitere respiratorische Symptome [1]. Das ZNS kann auch betroffen sein, als einziges annähernd pathognomonisches Symptom für COVID-19 wird der Verlust des Geruchs- und Geschmackssinns beschrieben, der bei einem Fünftel der Patienten in Deutschland beschrieben wurde [2, 3]. Epileptische Anfälle und Status epilepticus gehören zu den seltenen Manifestationen von COVID-19 [4]. Das Risiko für einen durch Komplikationen gekennzeichneten Verlauf und die Notwendigkeit einer stationären Behandlung hängen insbesondere vom Alter ab, aber auch bei Kindern und jungen Erwachsenen kann es zu schweren Verläufen mit Hospitalisierung und manchmal auch langer intensivmedizinischer Behandlung kommen [1].

Mit Epilepsie assoziierte Komorbiditäten wie das Down-Syndrom (Trisomie 21) [5] oder geistige Behinderung [6] können mit erheblich erhöhter Hospitalisierungsrate und Mortalität einhergehen. Die Bindung und Vorhaltung von Krankenhauskapazitäten haben zur Einschränkung in der allgemeinen Gesundheitsversorgung und auch in der Behandlung von Menschen mit Epilepsie geführt [7–10], sodass nur eine wirksame Bekämpfung der Pandemie zu einer Verbesserung der allgemeinen Gesundheitsversorgung führen kann. Neben wirksamen Schutzmaßnahmen gegen eine Ansteckung wie Händehygiene, Maskentragen und Distanzhalten kommt den neu entwickelten Impfstoffen gegen COVID-19 eine besondere Rolle zu, für die eine hohe Wirksamkeit in groß angelegten Studien gezeigt werden konnte [11–14]. Für den in Deutschland als ersten verfügbaren mRNA-Impfstoff Comirnaty® (BioNTech/Pfizer) konnte gezeigt werden, dass bis zu 95 von 100 geimpften Personen vollständig vor einer Erkrankung geschützt waren. Bei den übrigen 5 % kam es zu COVID-19 mit einem milden Verlauf, der keine Hospitalisierung erforderte [11]. Für den mRNA-Impfstoff von Moderna® wird eine vergleichbare Wirksamkeit von 94 % berichtet [12]. Somit ist die Impfung die wirksamste Maßnahme gegen COVID-19 und gegen seine bei einem Teil der Patienten auftretenden schweren oder langwierigen Komplikationen.

## Impfung und Epilepsie

Die Impfungen zur Vorbeugung der COVID-19-Erkrankung sind für Personen ab einem Alter von 16 Jahren zugelassen. Das Angebot zur Impfung erfolgt nach Vorgaben zur Priorisierung der Ständigen Impfkommission (STIKO) am Robert Koch-Institut [15] (s. unten).

Da bei einigen Menschen mit Epilepsie Verunsicherung besteht, ob diese Impfung zur Verschlechterung der Anfallssituation führen kann, haben wir eine Recherche zu den bereits verfügbaren und zu den weiteren wahrscheinlich bald zugelassenen Impfpräparaten durchgeführt. Die folgende Zusammenfassung basiert auf den Publikationen der Zulassungsstudien [11–14], den Fachinformationen der Europäischen Arzneimittelagentur (EMA; Summary of Product Characteristics) für die Impfstoffe Comirnaty® (Hersteller: BioNTech Manufacturing GmbH, Mainz, Deutschland) [16] und Moderna® (Hersteller: Rovi Pharma Industrial Services, S.A., Madrid, Spanien) [17] und denen der britischen Zulassungsbehörde (Medicines and Healthcare products Regulatory Agency) für die COVID-19 Vaccine AstraZeneca (ChAdOx1‑S; Hersteller: MedImmune UK Ltd, Liverpool, Vereinigtes Königreich) [18] (Details s. Tab. 1). Zum Zeitpunkt der Erstellung dieses Übersichtsartikels war sowohl die Bewertung der COVID-19 Vaccine AstraZeneca durch die EMA wie durch die STIKO noch ausstehend.

**Table 1:** 

*Impfstoff*	BNT162b2 mRNA Covid-19 Vaccine Comirnaty®	mRNA-1273 SARS-CoV‑2 Vaccine Moderna®	ChAdOx1 nCoV-19 vaccine (AZD1222)	Ad26.COV2.S Covid-19 Vaccine
*Entwickler* *Hersteller bzw. Vertrieb*	BioNTech/Pfizer	Moderna	Oxford University AstraZeneca	Johnson & Johnson
*Wirkungsweise*	mRNA-Impfstoff^b^	mRNA-Impfstoff^b^	Vektorimpfstoff^b^	Vektorimpfstoff^b^
*Wirksamkeit*	95 %	94 %	62–90 %	Keine Angabe
*Anzahl der Impfungen*	2 Dosen^a^ im Abstand von mindestens 21 Tagen	2 Dosen^a^ im Abstand von mindestens 28 Tagen	2 Dosen^a^ im Abstand zwischen 4 und 12 Wochen	Möglicherweise 1‑mal Gabe ausreichend
*Langfristige Lagerung*	−70 °C	−20 °C	Kühlschrank 2–8 °C	Keine Angabe
*Zulassung in der EU*	Dezember 2020 Ab 16 Jahren	Januar 2021 Ab 18 Jahren	Beantragt (in UK ab 18 Jahren zugelassen)	Keine Angabe

^a^Für den vollständigen Impfschutz und das Erreichen der oben genannten Wirksamkeit ist die Gabe beider Impfdosen notwendig

^b^mRNA- und Vektor-Impfstoffe verfolgen einen neuartigen Wirkungsweg und sind nicht als Lebendimpfstoffe anzusehen

Unter den bislang bekannten Nebenwirkungen sind in den genannten Publikationen und Fachinformationen epileptische Anfälle und Epilepsien nicht aufgeführt. Aktuell gibt es keine Hinweise darauf, dass für Menschen mit Epilepsie ein besonders hohes Risiko für Nebenwirkungen bei einer Impfung zur Vorbeugung der COVID-19-Erkrankung besteht. Nach allem verfügbaren Wissen ist für Menschen mit Epilepsie wie für die Allgemeinbevölkerung das Risiko eines ernsthaften Verlaufs von COVID-19 wesentlich höher als ein mögliches Risiko von Komplikationen bei Durchführung der Impfung.

Die Wirksamkeit der Impfung kann möglicherweise bei einer bestehenden Immunschwäche oder bei einer Behandlung, die die Immunantwort vermindert, beeinträchtigt sein. Hierzu zählen insbesondere Kortikosteroide (z. B. Prednisolon), Azathioprin oder auch monoklonale Antikörper wie Rituximab, die bei akut-symptomatischen Anfällen bei Autoimmunenzephalitis sowie autoimmun assoziierten Epilepsien [19] eingesetzt werden können. Dies gilt auch für den mTOR-Inhibitor Everolimus, der zur Zusatzbehandlung von epileptischen Anfällen im Zusammenhang mit tuberöser Sklerose eingesetzt werden kann [20]. In den genannten Fällen kann die Immunreaktion auf die Impfung möglicherweise beeinträchtigt und deshalb weniger wirksam sein [21]. Patienten, die immunsuppressiv behandelt werden, sollten das Ansprechen auf die Impfung und die Nutzen-Risiko-Abwägung mit dem behandelnden Arzt vor der Impfung erörtern. Das Risiko für einen schweren Verlauf einer COVID-19-Erkrankung scheint unter der chronischen Einnahme von immunsuppressiver Therapie nicht erhöht zu sein [22], im Idealfall sollten Impfungen 6 Wochen vor Beginn einer immunmodulierenden Behandlung durchgeführt werden. Die Autoren möchten zudem auf den ausführlichen Artikel „Impfen bei Immundefizienz“ verweisen [23].

Nach jeder Impfung kann es zu Fieber kommen, dies kann bei einigen Patienten mit Epilepsie anfallsauslösend wirken. Dieser anfallsprovozierende Faktor ist in der Regel bei den betroffenen Patienten durch vorherige fieberhafte Infekte oder Grippeschutzimpfungen bekannt. Auch die Impfstoffe zur Vorbeugung der COVID-19-Erkrankung können zu einer leichten Entzündungsreaktion mit Auftreten von Fieber führen. Hierauf wäre also nach einer Impfung zu achten, insbesondere wenn in der Vergangenheit in zeitlichem Zusammenhang mit Impfungen oder mit vorhergehenden Infekten epileptische Anfälle aufgetreten sind. Gegebenenfalls könnten fiebersenkende Mittel, die auch sonst von dem Patienten vertragen werden, z. B. Ibuprofen oder Paracetamol, eingesetzt werden [24]. Alternativ könnte vorübergehend die Dosis der Antiepileptika erhöht werden, oder es kann passager der Einsatz von Benzodiazepinen, wie z. B. Clobazam, erfolgen.

Die Fachinformationen der Impfstoffe sind zu beachten, insbesondere können Impfstoffe zur Vorbeugung der COVID-19-Erkrankung Inhaltsstoffe enthalten, gegen die eine Allergie bestehen kann. Sind Allergien und anaphylaktische Reaktionen in der Eigenanamnese bekannt, sollte dies mit dem die Impfung durchführenden Arzt besprochen werden.

## Impfpriorisierung

Nach Vorgaben der STIKO [15] erfolgt die Priorisierung des Impfangebotes für Personengruppen, bei denen ein hohes Risiko für schwere oder tödliche Verläufe einer COVID-19-Erkrankung vorliegt oder die beruflich entweder besonders exponiert sind oder engen Kontakt zu vulnerablen Personengruppen haben. Menschen mit epileptischen Anfällen oder Epilepsie werden in der STIKO-Empfehlung zur COVID-19-Impfung nicht als besondere Risikogruppe aufgeführt. Die Datenlage zur Gefährdung von Menschen mit Epilepsie durch eine COVID-19-Erkrankung ist nicht eindeutig, ohne begleitende Erkrankungen scheint kein erhöhtes Risiko zu bestehen. Bei Vorliegen von Begleiterkrankungen kann ein höheres Risiko bestehen [5, 6, 25].

Die bei Epilepsie möglicherweise auftretenden Komorbiditäten und die klinischen Manifestationen einer zugrunde liegenden Erkrankung können allerdings zu einer Priorisierung in eine höhere Stufe führen. Personen in Institutionen mit einer Demenz oder geistigen Behinderung sowie Personen mit einem Down-Syndrom (Trisomie 21) können der Stufe 2 (hohe Priorität), Personen z. B. mit Adipositas (BMI > 30) oder einer chronischen Nierenerkrankung können der Stufe 3 (erhöhte Priorität) und Personen z. B. mit schwerer Depression, chronischer Lebererkrankung, Immunkompromittierung, Diabetes mellitus, kardialer Arrhythmie/Vorhofflimmern, HIV-Infektion, koronarer Herzkrankheit, Herzinsuffizienz, zerebrovaskulären Erkrankungen/Schlaganfall, Autoimmunerkrankungen, COPD, Krebserkrankungen, arterieller Hypertonie, rheumatologischen Erkrankungen oder Asthma bronchiale können der Stufe 4 zugeordnet werden [15]. Auch für enge Kontaktpersonen von Schwangeren und enge Kontaktpersonen bzw. Pflegende von Personen mit hohem Risiko kann eine Priorisierung der Stufe 3 vorliegen.

Bei der Priorisierung innerhalb der COVID-19-Impfempfehlung der STIKO konnten nicht alle Krankheitsbilder oder Impfindikationen berücksichtigt werden [15], somit ist die Aufzählung nicht vollständig. Auch die Unterscheidung zwischen Personen mit Vorerkrankungen mit hohem Risiko (Stufe 3) gegenüber Vorerkrankungen mit moderat erhöhtem Risiko (Stufe 4) ist nicht immer eindeutig möglich. Deshalb sind Einzelfallentscheidungen möglich, die auch die Gesamtzahl der Vorerkrankungen und das Expositionsrisiko berücksichtigen. Es obliegt den für die Impfung Verantwortlichen, Personen, die nicht explizit genannt sind, in die jeweilige Priorisierungskategorie einzuordnen. Dies betrifft z. B. Menschen mit seltenen, schweren Vorerkrankungen, für die bislang zwar keine ausreichende wissenschaftliche Evidenz bezüglich des Verlaufes einer COVID-19-Erkrankung vorliegt, für die aber ein erhöhtes Risiko angenommen werden kann.

## Schlussfolgerung

Generell sollten Menschen mit Epilepsie den gleichen Impfschutz erhalten wie andere Menschen auch [26–28]. Zuvor in zeitlichem Zusammenhang mit Impfungen aufgetretene epileptische Anfälle sind keine Kontraindikation für eine Impfung zur Vorbeugung der COVID-19-Erkrankung [29]. In diesen seltenen Fällen ist es eine Einzelfallentscheidung, bei der Nutzen und Risiken zusammen mit dem behandelnden Arzt abgewogen werden müssen.

Eine generelle Priorisierung für die Impfung liegt bei Menschen mit Epilepsie nicht vor, relevante Komorbiditäten oder schwere Grunderkrankungen können jedoch zu einer Priorisierung führen. Die Autoren möchten in Namen der repräsentierten Fachgesellschaften Menschen mit Epilepsien ausdrücklich darin bestärken, falls keine Kontraindikationen bestehen, eine angebotene Impfung wahrzunehmen und sich und andere so vor einer schweren COVID-19-Erkrankung zu schützen.
